# Investigating the molecular control of deer antler extract on articular cartilage

**DOI:** 10.1186/s13018-020-02148-w

**Published:** 2021-01-06

**Authors:** Baojin Yao, Zhenwei Zhou, Mei Zhang, Xiangyang Leng, Daqing Zhao

**Affiliations:** 1 Jilin Ginseng Academy, Changchun University of Chinese Medicine, Changchun, 130117 China; 2 Innovation Practice Center, Changchun University of Chinese Medicine, Changchun, 130117 China; 3 The Affiliated Hospital of Changchun University of Chinese Medicine, Changchun, 130117 China

**Keywords:** Deer antler extract, Articular cartilage, RNA sequencing, Molecular mechanism, Therapeutic targets

## Abstract

**Background:**

Deer antler is considered as a precious traditional Chinese medicinal material and has been widely used to reinforce kidney’s yang, nourish essence, and strengthen bone function. The most prominent bioactive components in deer antler are water-soluble proteins that play potential roles in bone formation and repair. The aim of this study was to explore the molecular control and therapeutic targets of deer antler extract (DAE) on articular cartilage.

**Methods:**

DAE was prepared as previously described. All rats were randomly divided into Blank group and DAE group (10 rats per group) after 7-day adaptive feeding. The rats in DAE group were orally administrated with DAE at a dose of 0.2 g/kg per day for 3 weeks, and the rats in Blank group were fed with drinking water. Total RNA was isolated from the articular cartilage of knee joints. RNA sequencing (RNA-seq) experiment combined with quantitative real-time polymerase chain reaction (qRT-PCR) verification assay was carried out to explore the molecular control and therapeutic targets of DAE on articular cartilage.

**Results:**

We demonstrated that DAE significantly increased the expression levels of functional genes involved in cartilage formation, growth, and repair and decreased the expression levels of susceptibility genes involved in the pathophysiology of osteoarthritis.

**Conclusions:**

DAE might serve as a candidate supplement for maintaining cartilage homeostasis and preventing cartilage degeneration and inflammation. These effects were possibly achieved by accelerating the expression of functional genes involved in chondrocyte commitment, survival, proliferation, and differentiation and suppressing the expression of susceptibility genes involved in the pathophysiology of osteoarthritis. Thus, our findings will contribute towards deepening the knowledge about the molecular control and therapeutic targets of DAE on the treatment of cartilage-related diseases.

## Background

Deer antler is considered as a precious traditional Chinese medicinal material and has been widely used to reinforce kidney’s yang, nourish essence, and strengthen bone function [1]. The most prominent bioactive components in deer antler are water-soluble proteins that play potential roles in bone formation and repair [2–6]. However, little is known regarding their effects on cartilage development and growth. Deer antler is a fantastic mammalian appendage with characteristics of rapid growth and annually regeneration [7–9]. The longitudinal growth of the antler was considered as a modified endochondral ossification process that is analogous to the long bone growth originated from mesenchymal condensation, chondrocytic differentiation, and ossification [10]. During rapid growth stage, deer antler is capable to grow as rapidly as 2 cm per day, which represents the fastest rate of cartilaginous tissue growth in the mammal kingdom [11].

Antler growth is driven by the growth center that is located in the distal tip consisted of mesenchyme and cartilage tissues [12]. According to the application of deer antler in traditional Chinese medicine, the antler is divided into wax slice, powder slice, blood slice, and bone slice from distal tip to proximal base, and the wax slice is located in the distal tip of antler (growth center), which has the highest percentage of bioactive components [13, 14]. In our previous studies, we generated the deer antler extract (DAE), and the proportion of protein was 70% according to the protein concentration assay [15].

We also performed a series of analyses to investigate the effects of deer antler extract (DAE) on chondrocyte proliferation, differentiation, and apoptosis and demonstrated that freshly aqueous extracts from sika deer antlers at rapid growth stage could enhance chondrocyte viability and promote chondrocyte proliferation, but inhibit chondrocyte differentiation, maturation, and apoptosis. Furthermore, our results suggested that DAE play potential roles in boosting the abilities of chondrocytes against oxidative, inflammatory, and immune stresses [15, 16].

In the present study, we carried out state-of-the-art RNA sequencing (RNA-seq) experiment combined with quantitative real-time polymerase chain reaction (qRT-PCR) verification assay to explore the molecular control and therapeutic targets of DAE on articular cartilage. We demonstrated that DAE significantly increased the expression levels of functional genes involved in cartilage formation, growth, and repair and decreased the expression levels of susceptibility genes involved in the pathophysiology of osteoarthritis. Thus, DAE might serve as a candidate supplement for maintaining cartilage homeostasis and preventing cartilage degeneration and inflammation. These effects were possibly achieved by accelerating the expression of functional genes involved in chondrocyte commitment, survival, proliferation, and differentiation and suppressing the expression of susceptibility genes involved in the pathophysiology of osteoarthritis.

## Methods

### DAE preparation

The DAE used in the following experiments was the same as the ones that were prepared as previously described [15]. All experiments were approved by the Institutional Animal Ethics Committee of Changchun University of Chinese Medicine. Briefly, deer antlers in the rapid growth phase were obtained from three 4-year-old adult sika deers. The antlers were chopped into small pieces and thoroughly washed with ice water. The clean antler pieces were completely homogenized with a Tissue Homogenizer (Voshin, China) and centrifuged with an Eppendorf 5804R Refrigerated Centrifuge (Eppendorf, Germany). The supernatant was further clarified by filtering through a Hollow Fiber Membrane Filter Column (GE Healthcare, USA) and lyophilized with a Heto PowerDry LL3000 Freeze Dryer prior to storage at − 80 °C (Thermo, USA).

### Experimental animals

Twenty male Sprague-Dawley (SD) rats (SPF grade, 7 weeks old) were obtained from the Changchun Yisi laboratory animal technology Co, Ltd. (Changchun, China) with production license number SCXK (Ji) 2016-0003. The rats were housed with a constant temperature of 23 °C accompanied with a relative humidity of 50% in an air-conditioned room and exposed to a 12/12-h (light/dark) cycle. All animal protocols were approved by the Institutional Animal Care and Use Committee of Changchun University of Chinese Medicine, and all experimental procedures were performed in accordance with corresponding standards and guidelines.

### Drug administration and cartilage collection

All rats were randomly divided into Blank group and DAE group (10 rats per group) after 7-day adaptive feeding. The drug administration was carried out as previously described [5]. The rats in DAE group were orally administrated with DAE at a dose of 0.2 g/kg per day for 3 weeks, and the rats in Blank group were fed with drinking water. The administrated dose for DAE in rat experiment was calculated based on the body surface area normalization method [17]. Articular cartilage from each rat was harvested in the early morning after 3 weeks of DAE administration. Briefly, all rats were euthanized with CO_2_ inhalation and cervical dislocation to assure death. Articular cartilage from either the Blank group or the DAE group was carefully removed from the underlying subchondral bone from the left knee joint with a scalpel blade accompanied with a stereo microscope (Nikon, Japan) following Katagiri’s methods [18] and stored at − 80 °C for RNA extraction.

### RNA isolation and sequencing

Cartilage from each group was pooled together and pulverized into a powder in liquid nitrogen, respectively. Total RNA was isolated from the cartilage samples with the TRIzol reagent (Invitrogen, USA) according to the manufacturer’s instructions. The quality of RNA was assessed using an Agilent 2100 Bioanalyzer (Agilent Technologies, USA). Illumina 2 × 150 paired-end mRNA libraries were prepared with the TruSeq Stranded mRNA kit (Illumina, USA) according to the manufacturer’s instructions. Transcriptome sequencing was carried out by RNA-seq method on an Illumina HiSeq 2500 platform (Illumina, USA).

### RNA-seq data analysis

After RNA-seq, raw reads in FASTQ format were first processed by perl scripts. High-quality clean reads were generated by removing the low-quality reads and adapter sequences. The clean reads from each sample were aligned to the rat (*Rattus norvegicus*) reference genome via HISAT software [19]. Gene expression levels were evaluated by calculating the relative transcript abundance using the FPKM algorithm [20]. Genes with an FPKM smaller than 0.2 were considered not expressed and removed [21]. The transcripts were annotated using the BLAST program against the National Center for Biotechnology Information (NCBI) non-redundant (NR) and Swiss-Prot protein databases [22]. Differentially expressed genes (DEGs) between the Blank group and DAE group were identified using the DEGseq R package [23]. Transcripts with a log_2_ fold change ≥ 1 or ≤ − 1 and with a *p* value ≤ 0.001 were defined as DEGs. Gene ontology (GO) and Kyoto Encyclopedia of Genes and Genomes (KEGG) enrichment was analyzed using an R function phyper, and the hypergeometric test and Bonferroni correction were carried out for multiple testing corrections. GO terms or pathways with an adjusted *p* value (Q value) less than 0.05 were recognized to be significantly enriched [24].

### Verification of RNA-seq data by qRT-PCR

To verify the RNA-seq data, qRT-PCR was carried out to detect the expression levels of DEGs identified by RNA-seq analysis. Briefly, total RNA was isolated using TRIzol reagent (Invitrogen, USA) following the manufacturer’s instructions. cDNA synthesis was performed using an iScript cDNA Synthesis Kit (Bio-Rad, USA) and amplified using a SsoAdvanced Universal SYBR® Green Supermix (Bio-Rad, USA) on a CFX Connect Real-Time PCR Detection System (Bio-Rad, USA) under standard amplification conditions. Gene expression levels were calculated according to the 2^−ΔΔCT^ method by normalizing the rat glyceraldehyde 3-phosphate dehydrogenase gene (Gapdh) [25].

## Results

### Statistic summary of RNA-seq data

The messenger RNAs (mRNAs) of articular cartilage from rats with or without the treatment of DAE were sequenced using Illumina paired-end sequencing technology. The raw reads were uploaded in the NCBI Sequence Read Archive (SRA) database under BioProject accession number PRJNA642711. As shown in Table 1, 39,698,758 (Blank group) and 40,202,822 (DAE group) clean reads were obtained after trimming away the low-quality and adapter sequences, respectively, and the quality control results showed that the Q30 value was greater than 94%, and the GC content was around 51%. Therefore, the sequencing results were accurate and reliable for further analysis. By performing data mapping and annotation, 37,619,438 (Blank group) and 38,032,508 (DAE group) reads were mapped to the rat (*Rattus norvegicus*) genome; subsequently, 12,821 out of 15,664 (Blank group) and 12,902 out of 15,733 (DAE group) transcripts were obtained by annotating against the non-redundant (NR) NCBI protein database and Swiss-Prot database, respectively.

**Table 1:** Overview of output statistics of sequencing and assembly

Statistics	Blank	DAE
Clean reads	39,698,758	40,202,822
Q30 percentage	94.78	94.86
GC percentage	51.27	51.84
Total mapped reads	37,619,438	38,032,508
Total transcripts	15,664	15,733
Known transcripts	12,821	12,902

### DEGs identification, GO and KEGG enrichment analysis

**Fig. 1:**
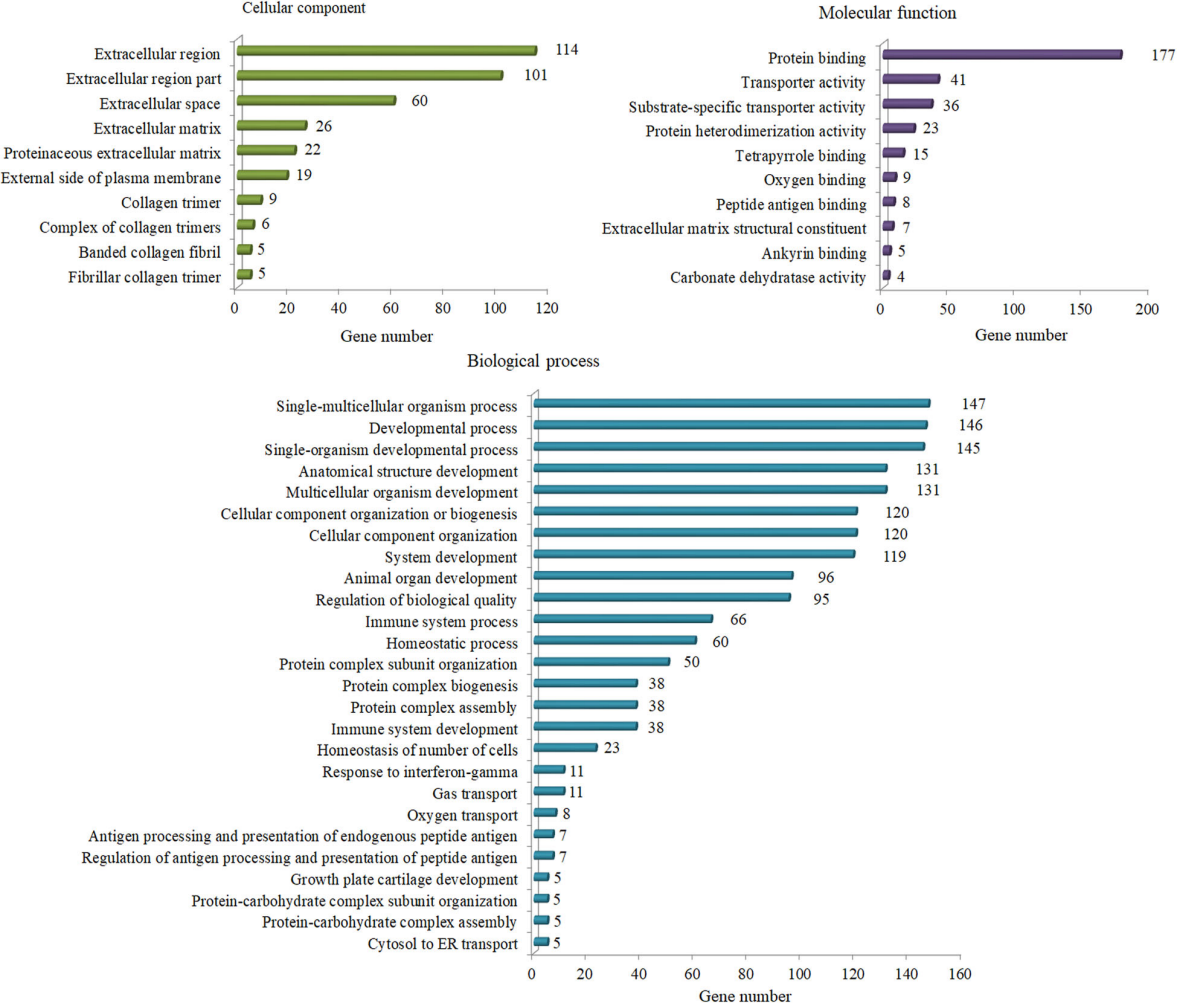
Histogram presentation of enriched GO terms in articular cartilage under DAE treatment. The horizontal coordinate represents the number of mapped genes in a category, and the vertical coordinate represents the GO terms significantly enriched (*p* < 0.05) in the categories of cellular component, molecular function, and biological process

**Fig. 2:**
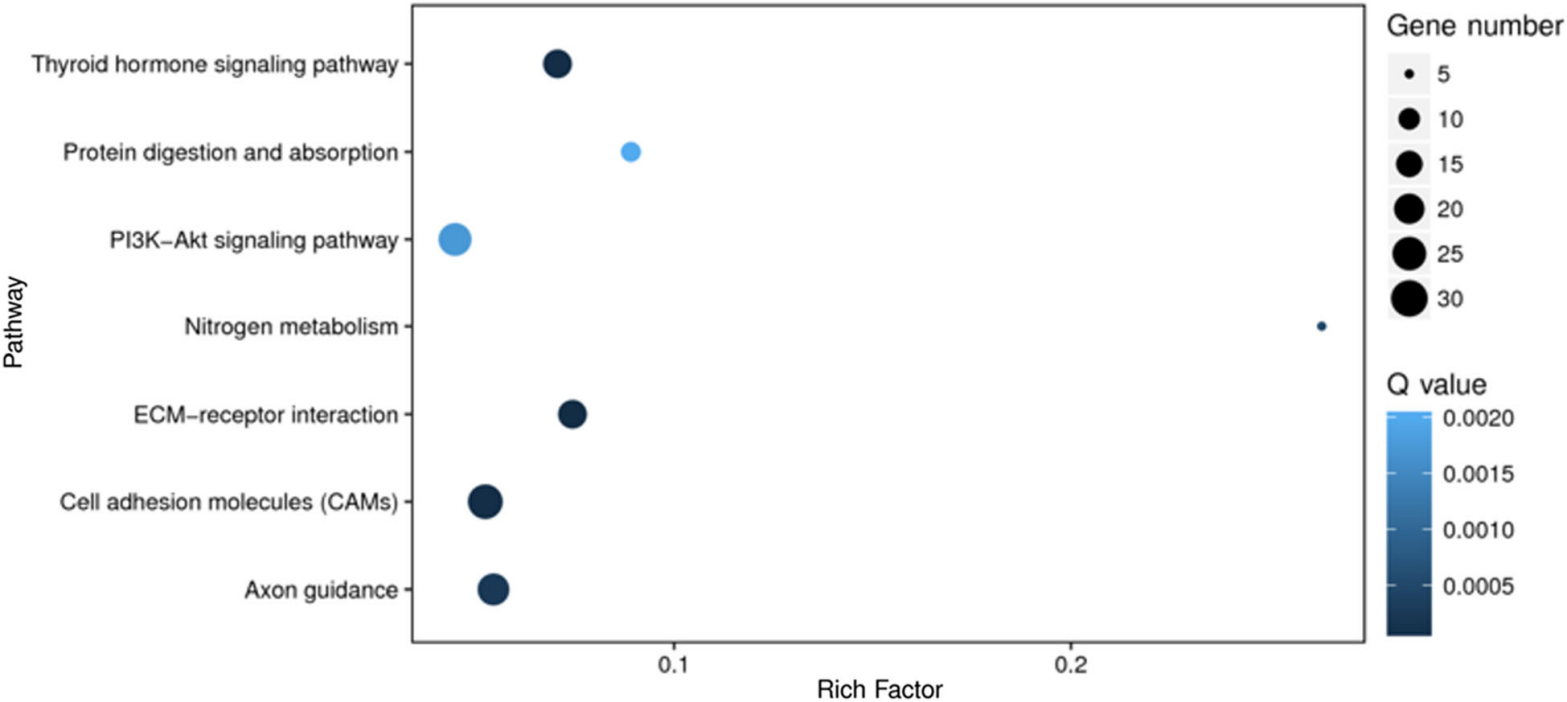
Scatter plot of enriched KEGG pathways in articular cartilage under DAE treatment. The horizontal coordinate represents the rich factor that is calculated as the ratio of the number of DEGs divided by the total gene number in a certain pathway, and the vertical coordinate represents the enriched pathway. The color and size of the dots represent the range of the Q values and the number of DEGs mapped to a certain pathway, respectively

By comparing the Blank group and DAE group, 308 genes were identified as DEGs under the established criteria (log_2_ fold change ≥ 1 or ≤ − 1 and *p* ≤ 0.001), including 208 upregulated genes (log_2_ fold change ≥ 1 and *p* ≤ 0.001) and 100 downregulated genes (log_2_ fold change ≤ − 1 and *p* ≤ 0.001), as shown in Table 2. GO enrichment analyses were carried out to gain insight into the biological functions of DEGs under DAE treatment, as shown in Fig. 1. Under the category of cellular component, the significantly enriched GO terms were mainly classified into extracellular region, extracellular region part, extracellular space, extracellular matrix, and proteinaceous extracellular matrix; under the category of molecular function, the significantly enriched GO terms were mainly classified into protein binding, transporter activity, substrate-specific transporter activity, protein heterodimerization activity, and tetrapyrrole binding; under the category of biological process, the significantly enriched GO terms were mainly classified into single-multicellular organism process, developmental process, single-organism developmental process, anatomical structure development, and multicellular organism development. KEGG pathway enrichment analyses were carried out to further explore the possible functional pathways of DEGs under DAE treatment, as shown in Fig. 2; the significant enriched pathways were predominantly mapped to thyroid hormone signaling pathway, protein digestion and absorption, PI3K-AKT signaling pathway, nitrogen metabolism, ECM-receptor interaction, and cell adhesion molecules (CAMs).

**Table 2:** Statistics for the differentially expressed genes (DAE vs. Blank)

Statistics	Number
Differentially expressed mRNAs	308
Upregulated mRNAs	208
Downregulated mRNAs	100

### DAE significantly increased the expression levels of functional genes involved in cartilage formation, growth, and repair

According to the analysis of DEGs, 36 genes involved in cartilage formation, growth, and repair were identified, such as hyaluronan and proteoglycan link protein 1 (Hapln1), collagen alpha-1(IX) chain (Col9a1), scrapie-responsive protein 1 (Scrg1), cartilage oligomeric matrix protein (Comp), leukocyte cell-derived chemotaxin 1 (Cnmd), carbonic anhydrase 2 (Ca2), matrilin-3 (Matn3), transferrin receptor protein 1 (Tfrc), vascular endothelial growth factor A (Vegfa), and collagen alpha-1(XXVII) chain (Col27a1), as shown in Table 3.

**Table 3:** List of significantly upregulated DEGs involved in cartilage formation, growth, and repair

Gene name	Expression level (FPKM)		log_2_ fold change (DAE/Blank)	*p* value
	Blank	DAE		
Hyaluronan and proteoglycan link protein 1 (Hapln1)	301.97	901.64	1.58	0
Collagen alpha-1(IX) chain (Col9a1)	142.16	519.76	1.87	0
Scrapie-responsive protein 1 (Scrg1)	203.52	491.32	1.27	1.23E−92
Cartilage oligomeric matrix protein (Comp)	230.34	490.17	1.09	0
Leukocyte cell-derived chemotaxin 1 (Cnmd)	135.34	397.34	1.55	1.08e−321
Carbonic anhydrase 2 (Ca2)	172.19	352.43	1.03	2.61E−159
Matrilin-3 (Matn3)	112.11	292.66	1.38	0
Transferrin receptor protein 1 (Tfrc)	46.77	117.84	1.33	1.65E−274
Vascular endothelial growth factor A (Vegfa)	51.06	107.19	1.07	8.57E−143
Collagen alpha-1(XXVII) chain (Col27a1)	41.63	90.66	1.12	2.44E−248
M-phase inducer phosphatase 2 (Cdc25b)	33.93	76.00	1.16	1.82E−92
Proliferation marker protein Ki-67 (Mki67)	21.76	45.38	1.06	6.36E−136
Carbonic anhydrase 12 (Ca12)	10.53	35.95	1.77	2.03E−95
Radical S-adenosyl methionine domain-containing protein 2 (Rsad2)	9.91	29.91	1.59	1.27E−69
T cell acute lymphocytic leukemia protein 1 homolog (Tal1)	10.64	23.50	1.14	2.07E−44
Chondroitin sulfate *N*-acetylgalactosaminyltransferase 1 (Csgalnact1)	11.15	22.80	1.03	3.03E−18
Cartilage matrix protein (Matn1)	4.78	21.71	2.18	3.49E−40
Chordin-like protein 1 (Chrdl1)	7.49	19.15	1.35	2.56E−41
Thrombospondin-3 (Thbs3)	5.25	16.71	1.67	7.21E−37
Complement decay-accelerating factor, GPI-anchored (Cd55)	7.74	15.89	1.04	1.04E−10
Mitotic checkpoint serine/threonine-protein kinase BUB1 (Bub1)	7.32	14.81	1.02	4.70E−23
G1/S-specific cyclin-E2 (Ccne2)	6.42	14.00	1.12	1.48E−17
Protein MGARP (Mgarp)	4.50	12.66	1.49	1.12E−09
M-phase phosphoprotein 8 (Mphosph8)	5.78	12.46	1.11	2.24E−14
Noggin (Nog)	4.80	11.69	1.28	7.67E−10
Carbonic anhydrase 9 (Ca9)	5.45	11.63	1.09	7.97E−10
ESF1 homolog (Esf1)	5.27	11.61	1.14	1.35E−16
Aryl hydrocarbon receptor nuclear translocator-like protein 1 (Arntl)	3.14	10.98	1.81	2.48E−25
H(+)/Cl(-) exchange transporter 3 (Clcn3)	4.15	9.18	1.15	1.12E−08
G2/M phase-specific E3 ubiquitin-protein ligase (G2e3)	3.80	7.66	1.01	2.15E−13
Dual specificity tyrosine-phosphorylation-regulated kinase 3 (Dyrk3)	2.45	6.59	1.43	7.06E−09
Protein kinase C zeta type (Prkcz)	2.98	6.32	1.08	5.86E−07
WNT1-inducible-signaling pathway protein 3 (Wisp3)	2.82	5.89	1.06	2.27E−06
Centrosomal protein of 70 kDa (Cep70)	2.02	4.72	1.22	2.28E−08
Dermatopontin (Dpt)	1.46	3.97	1.44	3.09E−07
Murinoglobulin-1 (Mug1)	0.56	1.67	1.58	3.76E−06

### DAE significantly decreased the expression levels of susceptibility genes involved in the pathophysiology of osteoarthritis

According to the analysis of DEGs, 31 genes involved in osteoarthritis susceptibility were identified, such as collagen alpha-1(I) chain (Col1a1), protein S100-A4 (S100a4), fatty acid-binding protein (Fabp4), collagen alpha-1(IV) chain (Col4a1), cyclic AMP-responsive element-binding protein 3-like protein 1 (Creb3l1), C-C motif chemokine 9 (Ccl9), retinoid-binding protein 7 (Rbp7), neurogenic locus notch homolog protein 3 (Notch3), C-X-C motif chemokine 16 (Cxcl16), and receptor activity-modifying protein 3 (Ramp3), as shown in Table 4.

**Table 4:** List of significantly downregulated DEGs involved in osteoarthritis susceptibility

Gene name	Expression level (FPKM)		log_2_ fold change (DAE/Blank)	*p* value
	Blank	DAE		
Collagen alpha-1(I) chain (Col1a1)	6244.47	2741.45	− 1.19	0
Protein S100-A4 (S100a4)	687.97	330.60	− 1.06	2.85E−77
Fatty acid-binding protein (Fabp4)	150.74	74.48	− 1.02	1.22E−22
Collagen alpha-1(IV) chain (Col4a1)	60.42	26.01	− 1.22	1.55E−166
Cyclic AMP-responsive element-binding protein 3-like protein 1 (Creb3l1)	53.52	23.54	− 1.18	1.14E−56
C-C motif chemokine 9 (Ccl9)	34.51	11.42	− 1.60	2.15E−27
Retinoid-binding protein 7 (Rbp7)	19.22	5.96	− 1.69	1.21E−07
Neurogenic locus notch homolog protein 3 (Notch3)	18.11	8.55	− 1.08	1.69E−47
C-X-C motif chemokine 16 (Cxcl16)	17.30	7.62	− 1.18	1.19E−12
Receptor activity-modifying protein 3 (Ramp3)	16.14	6.77	− 1.25	2.83E−09
Cytochrome P450 1B1 (Cyp1b1)	15.63	6.84	− 1.19	1.50E−15
Proprotein convertase subtilisin/kexin type 1 inhibitor (Pcsk1n)	15.53	6.80	− 1.19	1.73E−06
Homeobox protein DLX-3 (Dlx3)	15.49	7.31	− 1.08	3.27E−15
Protein naked cuticle homolog 2 (Nkd2)	14.53	6.59	− 1.14	1.00E−22
Matrix metalloproteinase-19 (Mmp19)	12.71	6.07	− 1.07	2.53E−09
Endosialin (Cd248)	11.69	5.06	− 1.21	2.00E−13
Pentraxin-related protein PTX3 (Ptx3)	10.83	5.32	− 1.03	1.16E−07
Fc receptor-like protein 2 (Fcrl2)	10.76	4.82	− 1.16	1.76E−10
Serine protease HTR4 (Htra4)	10.74	4.32	− 1.31	2.14E−12
Transforming growth factor beta-1-induced transcript 1 protein (Tgfb1i1)	10.61	5.05	− 1.07	3.56E−08
Apelin receptor (Aplnr)	10.59	3.76	− 1.49	2.16E−23
Tubulin beta-2B chain (Tubb2b)	7.26	3.04	− 1.26	1.80E−06
Arginase-1 (Arg1)	5.65	2.37	− 1.25	8.66E−05
Nostrin (Nostrin)	5.43	2.16	− 1.33	1.80E−05
von Willebrand factor (Vwf)	3.76	1.63	− 1.21	3.03E−15
Tenascin-X (Tnxb)	2.82	1.27	− 1.15	4.95E−05
Aryl hydrocarbon receptor (Ahr)	2.41	1.16	− 1.05	1.62E−05
Interferon-induced protein with tetratricopeptide repeats 1 (Ifit1)	2.01	0.54	− 1.90	1.02E−04
Interleukin-2 receptor subunit beta (Il2rb)	1.91	0.59	− 1.69	1.31E−04
Chloride intracellular channel protein 5 (Clic5)	1.81	0.51	− 1.83	1.62E−09
C-type lectin domain family 9 member A (Clec9a)	1.47	0.43	− 1.77	9.17E−05

### Gene expression levels of DEGs validated by qRT-PCR

**Fig. 3:**
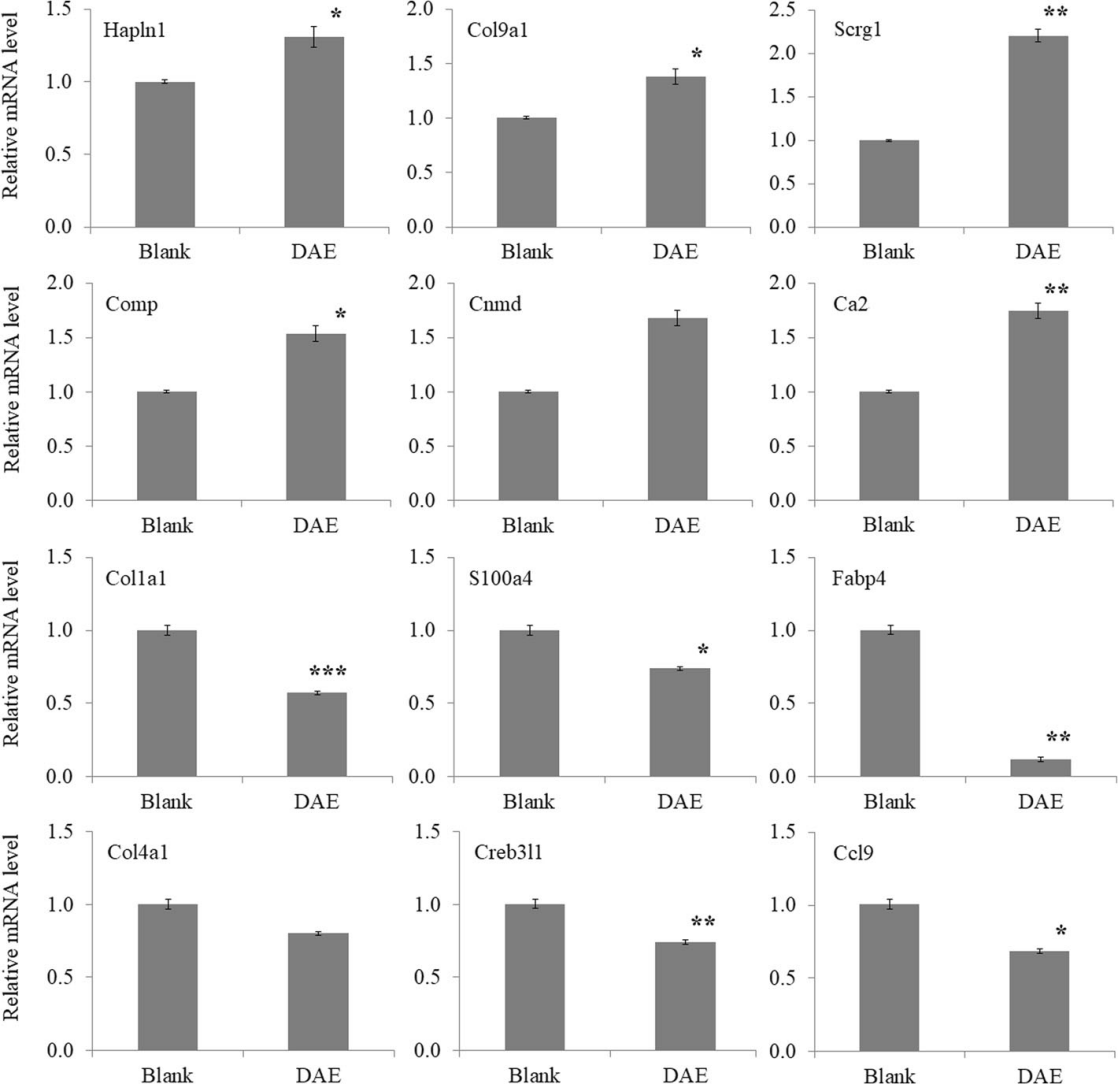
qRT-PCR validation of RNA-seq data. Data shown are representative of multiple independent experiments (*n* = 3) with biological triplicates per experiment with their corresponding standard deviation. The asterisk *, **, and *** indicate significant differences using the Student *t* test with *p* value < 0.05, < 0.01, and < 0.001, respectively. Gene expression levels for individual genes are presented as the ratio of (fold change) the DAE group to the Blank group

The expression levels of a series of DEGs were validated by qRT-PCR assay, including 6 significantly upregulated genes and 6 significantly downregulated genes. The specific gene primers for qRT-PCR were listed in Table 5. The relative fold change of each gene was normalized to the internal reference gene Gapdh. The expression levels of the selected DEGs validated by qRT-PCR exhibited similar expression patterns as those of the RNA-seq analysis, as shown in Fig. 3.

**Table 5:** List of primers used for qRT-PCR validation

Gene	Primer	Sequence
Hapln1	Forward primer	GACAGCTACACTCCGGATCA
	Reverse primer	AGCCAAATGCTGTAGGGTCT
Col9a1	Forward primer	CCAGCACATCAAGCAGGTTT
	Reverse primer	CCTCCCAGGAAGACCAGAAG
Scrg1	Forward primer	CCTTGTCATCCTCGGGCTAA
	Reverse primer	AACAGGAGAGGCGACTTGAA
Comp	Forward primer	CAGCTCAAGGCTGTCAAGTC
	Reverse primer	CTTCCAGCCCACATTTCGAG
Cnmd	Forward primer	TTACCACCAGCAGGAAGGAG
	Reverse primer	TGAGCGACACCCTTGGTAAT
Ca2	Forward primer	TCCTTGCTCCCTTCTTCCTG
	Reverse primer	CAGGTCACACATTCCAGCAG
Col1a1	Forward primer	ACCTCAGGGTATTGCTGGAC
	Reverse primer	GACCAGGGAAGCCTCTTTCT
S100a4	Forward primer	GCTGCATTCCAGAAGCTGAT
	Reverse primer	CATCATGGCAATGCAGGACA
Fabp4	Forward primer	ATGTGCAGAAGTGGGATGGA
	Reverse primer	GTCACGCCTTTCATGACACA
Col4a1	Forward primer	CCTCCAGGAACGACTACTCC
	Reverse primer	GCACACCTGCTAATGAAGGG
Creb3l1	Forward primer	CAGCTGCAGAAACTCCAGAC
	Reverse primer	CCAGAACAAAGCACAAGGCT
Ccl9	Forward primer	TCGTACATGCGACAGAGACA
	Reverse primer	TGGACCCGTGAGGTATAGGA

## Discussion

For many years, RNA-seq has become an essential tool for studying the dynamic and complex characteristics of the transcriptome, especially for the mRNA molecules, which encode proteins following the central dogma of molecular biology [26, 27]. Meanwhile, many scientists in the research field of Chinese medicine have paid more and more attention to explore the molecular control of Chinese herbs and formulations by taking advantage of RNA-seq technology [28, 29]. Our previous studies have shown that DAE play potential roles in regulating chondrocyte proliferation and differentiation [15, 16]. Therefore, in the present study, we further investigated the effects of DAE on articular cartilage using a state-of-the-art RNA-seq technology accompanied with validation method to obtain the precise molecular mechanism of DAE on cartilage homeostasis.

In total, 308 DEGs were identified, including 208 upregulated genes and 100 downregulated genes by comparing DAE-treated group with Blank group (DAE vs. Blank). According to the GO enrichment analysis, the significantly enriched GO terms were predominantly involved in extracellular matrix synthesis, binding activity, and developmental process. Those functional gene groups play pivotal roles in regulating cartilage homeostasis [30–32]. Based on the KEGG enrichment analysis, the significantly enriched pathways were predominantly involved in thyroid hormone signaling pathway, protein digestion and absorption, PI3K-AKT signaling pathway, nitrogen metabolism, ECM-receptor interaction, and cell adhesion molecules (CAMs). Among those enriched signaling pathways, thyroid hormone signaling pathway, PI3K-AKT signaling pathway, ECM-receptor interaction, and cell adhesion molecules (CAMs) have been considered to play crucial roles in articular cartilage maintenance and osteoarthritis pathogenesis [33–36]. Thus, these results suggest that DAE play potential role in regulating articular cartilage homeostasis by controling multiple functional group genes and signaling pathways.

Among the significantly upregulated DEGs under DAE treatment, 36 genes that participate in cartilage formation, growth, and repair were identified. For instance, Hapln1, Col9a1, Comp, Cnmd, Matn3, Col27a1, Matn1, and Dpt are extracellular matrixes that play key roles in regulating chondrocyte metabolism and functions via cell-matrix interaction [37–41]. Scrg1 is a stimulator of chondrogenesis that has a potential role in tissue engineering of articular cartilage [42]. Tfrc, also known as CD71, is a transferrin receptor that is essential for cartilage maturation during embryonic development [43]. Vegfa, a member of the Vegf growth factor family, is a key component to support chondrocyte survival [44]. Ca2, Ca12, and Ca9 are family members of carbonic anhydrases, which are important for cartilage homeostasis. All of them are significantly expressed in the articular chondrocytes, and Ca2 is mainly localized in the proliferating chondrocytes [45]. Rsad2, also known as viperin, is highly expressed in the middle zone of articular cartilage [46]. Tal1 is a basic helix-loop-helix transcription factor in the articular chondrocytes and serves as a crucial regulator during chondrocyte maturation [47].

Csgalnact1 is a glycosyltransferase that is necessary for the biosynthesis of chondroitin sulfate proteoglycans in cartilage, particularly in the proliferating chondrocytes [48]. Chrdl1, a secreted glycoprotein, is considered to be a juvenile chondrocyte-specific factor that stimulates stem cell growth [49]. Thbs3 is mainly expressed in the proliferating chondrocytes and inhibits cartilage clarification [50]. Cd55 is a complement decay-accelerating factor in the cartilage that plays pivotal role in protecting chondrocytes from possible damage [51]. Nog serves as an inhibitor of bone morphogenetic proteins (BMPs) and prevents cartilage degeneration and osteoarthritis development by inhibiting Il1β and Bmp2 expression [52]. Esf1 is an essential nucleolar protein that is required for cartilage formation [53]. Arntl, also known as Bmal1, plays crucial role in controling cartilage homeostasis through modulating TGF-β signaling [54]. Clcn3 is a highly expressed channel protein in chondrocytes during cartilage development and plays a key role in cell volume regulation [55]. Wisp3 is a multi-domain protein that maintains cartilage integrity and prevents chondrocyte hypertrophy [56]. Mug1 serves as an inhibitor of proteolytic enzyme that prevents the degradation of cartilage extracellular matrix [57]. In addition, Cdc25b, Mki67, Bub1, Ccne2, Mphosph8, G2e3, Dyrk3, and Cep70 are considered to be essential genes involved in cell proliferation [58–64]. Thus, these results suggest that DAE might serve as a candidate supplement for maintaining cartilage homeostasis.

Among the significantly downregulated DEGs under DAE treatment, 31 genes involved in osteoarthritis susceptibility were identified. For instance, Col1a1 and Col4a1 are collagen fibers that were associated with the progression of osteoarthritis [65]. S100a4, a member of the S100 protein family, is involved in cartilage degradation of osteoarthritis pathophysiology [66]. Fabp4 is a fatty acid-binding protein that serves as a biomarker for knee osteoarthritis [67]. Creb3l1 is a transcription factor significantly upregulated at the early stage of osteoarthritis [68]. Ccl9, also called macrophage inflammatory protein-1 gamma (MIP-1γ), is a small cytokine belonging to the CC chemokine family that was shown to be highly expressed during osteoarthritis progression [69]. Rbp7, a family member of the cellular retinol-binding proteins, is significantly upregulated in osteoarthritic chondrocytes [70]. Notch3 is a family member of Notch receptors, and genetic deletion of Notch3 or the blockade of Notch3 signaling prevents joint damage and attenuates inflammation of inflammatory arthritis [71].

Cxcl16 (a chemokine ligand), Cd248 (a transmembrane glycoprotein), Ptx3 (an inflammation induced gene), Fcrl2 (a subtype of Fc receptor-like molecules), Tgfb1i1 (a transforming growth factor beta 1 induced gene), Nostrin (a nitric oxide synthase traffic inducer), Vwf (an adhesive and multimeric glycoprotein), Tnxb (a member of the tenascin family), Ahr (a ligand-activated transcription factor), Ifit1 (an interferon-induced protein), Il2rb (an interleukin-2 receptor subunit), and Clec9a (a C-type lectin) are involved in the inflammation associated with arthritis pathology [72–83]. Ramp3 is a receptor activity-modifying protein that is highly expressed during joint inflammation [84]. Cyp1b1 (a member of the cytochrome P450 enzyme family), Pcsk1n (an inhibitor of prohormone convertase 1), Dlx3 (a family member of homeobox proteins), Nkd2 (a regulator of inflammatory response), Mmp19 (a subtype of matrix metalloproteinases), Htra4 (a subtype of serine proteases), Aplnr (a G protein-coupled receptor of apelin), Tubb2b (a beta isoform of tubulin), Arg1 (a cytosolic manganese-dependent enzyme), and Clic5 (a chloride intracellular channel protein) are reported to be highly expressed under osteoarthritic condition [85–94]. Thus, these results suggest that DAE might serve as a candidate supplement for preventing cartilage degeneration and inflammation.

In addition to the above findings, we also compared the expression levels of genes that are well known to characterize hyaline cartilage, such as Sox9, Sox5, Sox6, Wwp2, Acan, Col2a1, Col9a1, Col11a1, Hapln1, Comp, Matn1, Ptch1, Fgfr3, Runx2, and Runx3. As shown in Table S1, the expression levels of a majority of these genes were slightly upregulated under the DAE treatment. However, the expression level of Sox9 was slightly downregulated, which indicates that DAE might regulate articular chondrocytes through other transcription factors and related signaling pathways.

Nevertheless, there are still some limitations in the present study. First, this study lacks pathological analysis of animal model by performing the histological, behavioral, and biochemical evaluations; further experiments in the future still need to be carried out to investigate the effects of DAE on osteoarthritis model to get insight into their exact roles in regulating cartilage repair and regeneration. Second, this study included only a small sample size, although it is very difficult to obtain enough qualified articular cartilage from rats, which largely limits the sample size. However, more sample sizes still need to be included in future studies, and it is better to have at least 3 to 5 independent tissue samples be independently assessed for statistical analysis of a molecular outcome.

## Conclusion

In summary, the present study demonstrated that DAE might serve as a candidate supplement for maintaining cartilage homeostasis and preventing cartilage degeneration and inflammation. These effects were possibly achieved by accelerating the expression of functional genes involved in cartilage formation, growth, and repair and suppressing the expression of susceptibility genes involved in the pathophysiology of osteoarthritis. Thus, our findings will contribute towards deepening the knowledge about the molecular control and therapeutic targets of DAE on the treatment of cartilage-related diseases.

## Supplementary Information

**Additional file 1: Table S1.**

## Data Availability

The datasets used and/or analyzed during the current study are available from the corresponding author on reasonable request.

## Abbreviations

DAE: Deer antler extract
SD: Sprague-Dawley
RNA-seq: RNA sequencing
GO: Gene ontology
KEGG: Kyoto Encyclopedia of Genes and Genomes
DEGs: Differentially expressed genes
NCBI: National Center for Biotechnology Information
NR: Non-redundant
SRA: Sequence Read Archive
qRT-PCR: Quantitative real-time PCR
Gapdh: Glyceraldehyde 3-phosphate dehydrogenase
mRNAs: Messenger RNAs
