# Supplementary material for: Investigating the molecular control of deer antler extract on articular cartilage
Source: J Orthop Surg Res. 2021 Jan 6;16:8. doi: 10.1186/s13018-020-02148-w (PMC7788833; doi:10.1186/s13018-020-02148-w)
Supplement: Supplementary file 1 — Additional file 1: Table S1. [file 13018_2020_2148_MOESM1_ESM.doc]

**Table S1 List of well-known genes in hyaline cartilage**

| Gene name | Expression level (FPKM) | | log_2_ fold change (DAE/Blank) | p value |
| --- | --- | --- | --- | --- |
|  | Blank | DAE |  |  |
| Transcription factor SOX-9 (Sox9) | 27.10 | 22.63 | -0.26 | 3.72E-04 |
| Transcription factor SOX-5 (Sox5) | 4.23 | 5.25 | 0.31 | 4.80E-04 |
| Transcription factor SOX-6 (Sox6) | 5.06 | 9.81 | 0.96 | 1.36E-14 |
| NEDD4-like E3 ubiquitin-protein ligase WWP2 (Wwp2) | 84.81 | 100.26 | 0.24 | 8.52E-10 |
| Aggrecan core protein (Acan) | 417.31 | 542.77 | 0.38 | 4.25E-234 |
| Collagen alpha-1(II) chain (Col2a1) | 7243.17 | 11927.60 | 0.72 | 0 |
| Collagen alpha-1(IX) (Col9a1) | 142.16 | 519.76 | 1.87 | 0 |
| Collagen alpha-1(XI) (Col11a1) | 195.41 | 354.06 | 0.86 | 0 |
| Hyaluronan and proteoglycan link protein 1 (Hapln1) | 301.97 | 901.64 | 1.58 | 0 |
| Cartilage oligomeric matrix protein (Comp) | 230.34 | 490.17 | 1.09 | 0 |
| Cartilage matrix protein (Matn1) | 4.78 | 21.71 | 2.18 | 3.49E-40 |
| Protein patched homolog 1 (Ptch1) | 19.05 | 26.28 | 0.46 | 1.23E-16 |
| Fibroblast growth factor receptor 3 (Fgfr3) | 40.95 | 59.68 | 0.54 | 1.04E-23 |
| Runt-related transcription factor 2 (Runx2) | 18.69 | 20.26 | 0.12 | 7.18E-02 |
| Runt-related transcription factor 3 (Runx3) | 19.70 | 20.31 | 0.04 | 3.66E-01 |
